# LINE-1 transcription in round spermatids is associated with accretion of 5-carboxylcytosine in their open reading frames

**DOI:** 10.1038/s42003-021-02217-8

**Published:** 2021-06-07

**Authors:** Martin J. Blythe, Ayhan Kocer, Alejandro Rubio-Roldan, Tom Giles, Abdulkadir Abakir, Côme Ialy-Radio, Lee M. Wheldon, Oxana Bereshchenko, Stefano Bruscoli, Alexander Kondrashov, Joël R. Drevet, Richard D. Emes, Andrew D. Johnson, John R. McCarrey, Daniel Gackowski, Ryszard Olinski, Julie Cocquet, Jose L. Garcia-Perez, Alexey Ruzov

**Affiliations:** 1 Deep Seq, University of Nottingham, Queen’s Medical Centre, Nottingham, UK; 2 GReD Laboratory, CNRS UMR 6293 - INSERM U1103 - Clermont Université, Aubière, France; 3 GENYO, Centre for Genomics and Oncological Research, Pfizer/University of Granada/Andalusian Regional Government, PTS Granada, Granada, Spain; 4 Digital Research Service, Sutton Bonington Campus, University of Nottingham, Sutton Bonington, Leicestershire UK; 5 School of Medicine, University of Nottingham, University Park, Nottingham, UK; 6 INSERM U1016, Institut Cochin - CNRS UMR8104 - Faculté de Médecine, Université Paris Descartes, Sorbonne Paris Cité, Paris, France; 7 Medical Molecular Sciences, University of Nottingham, University Park, Nottingham, UK; 8 Department of Medicine, Section of Pharmacology, University of Perugia, Perugia, Italy; 9 School of Veterinary Medicine and Science, Sutton Bonington Campus, University of Nottingham, Sutton Bonington, Leicestershire UK; 10 School of Life Sciences, University of Nottingham, University Park, Nottingham, UK; 11 University of Texas at San Antonio, San Antonio, TX USA; 12 Department of Clinical Biochemistry, Collegium Medicum, Nicolaus Copernicus University, Bydgoszcz, Poland; 13 MRC Human Genetics Unit, Institute of Genetics and Molecular Medicine, University of Edinburgh, Edinburgh, UK

**Keywords:** DNA methylation, Spermatogenesis

## Abstract

Chromatin of male and female gametes undergoes a number of reprogramming events during the transition from germ cell to embryonic developmental programs. Although the rearrangement of DNA methylation patterns occurring in the zygote has been extensively characterized, little is known about the dynamics of DNA modifications during spermatid maturation. Here, we demonstrate that the dynamics of 5-carboxylcytosine (5caC) correlate with active transcription of LINE-1 retroelements during murine spermiogenesis. We show that the open reading frames of active and evolutionary young LINE-1s are 5caC-enriched in round spermatids and 5caC is eliminated from LINE-1s and spermiogenesis-specific genes during spermatid maturation, being simultaneously retained at promoters and introns of developmental genes. Our results reveal an association of 5caC with activity of LINE-1 retrotransposons suggesting a potential direct role for this DNA modification in fine regulation of their transcription.

## Abstract (web summary)

Blythe et al investigate the patterns of oxidized forms of the 5-methylcytosine DNA modification during spermatogenesis. They find that open reading frames of evolutionarily young and transcriptionally active LINE-1 retrotransposons are enriched in 5-carboxylcytosine (5caC) during spermatid maturation, suggesting a link between 5cac and LINE-1 activity.

## Introduction

The chromatin of both maternal and paternal pronuclei of the mammalian zygote undergoes extensive genome-wide reprogramming after fertilization, as the embryo transitions from germ cell to somatic developmental programs^1^. This process involves reorganization of the patterns of 5-methylcytosine (5mC), a DNA modification associated with regulation of gene activity^1,2^ and repression of transposable elements (TEs)^3^. Moreover, both maternal and paternal genomes are subjected to TET3-dependent oxidation of 5mC to 5-hydroxymethylcytosine (5hmC), 5-formylcytosine (5fC), and 5-carboxylcytosine (5caC) in one-cell embryos^4–7^. Although the precise biological functions of these oxidized forms of 5mC remain elusive, they likely contribute to the embryonic developmental program due to their anticipated roles in DNA demethylation^8^ and transcriptional regulation^9–11^.

In contrast with a large volume of experimental data on the rearrangement of DNA methylation patterns in pre-implantation embryos, little is known about the dynamics of DNA modifications during spermatid maturation. Nevertheless, a number of studies imply different epigenetic states for round spermatids (rST) and spermatozoa (SZ) nuclei^12,13^. Thus, the rate of successful development of the embryos produced by rST injection into oocytes (ROSI) is substantially lower than that of the embryos generated by injection using mature spermatozoa (intracytoplasmic sperm injection, ICSI). Moreover, ROSI-derived embryos exhibit aberrant patterns of zygotic DNA methylation^13^ and impaired zygotic demethylation^12^.

Since, 5fC/5caC can be recognized and excised from DNA by thymine-DNA glycosylase (TDG) followed by subsequent incorporation of unmodified cytosine into the abasic site by the components of the base excision repair (BER) pathway^8^, transient accumulation of these marks during differentiation may serve as an indicator of active demethylation^14,15^.

In this study, we aimed to examine whether TDG/BER-dependent demethylation is utilized in spermatogenesis and to determine the patterns of the genomic distribution of oxidized forms of 5mC during spermatid maturation.

## Results

### The levels of 5caC substantially decrease in testis germ cells during spermatid maturation

**Fig. 1:**
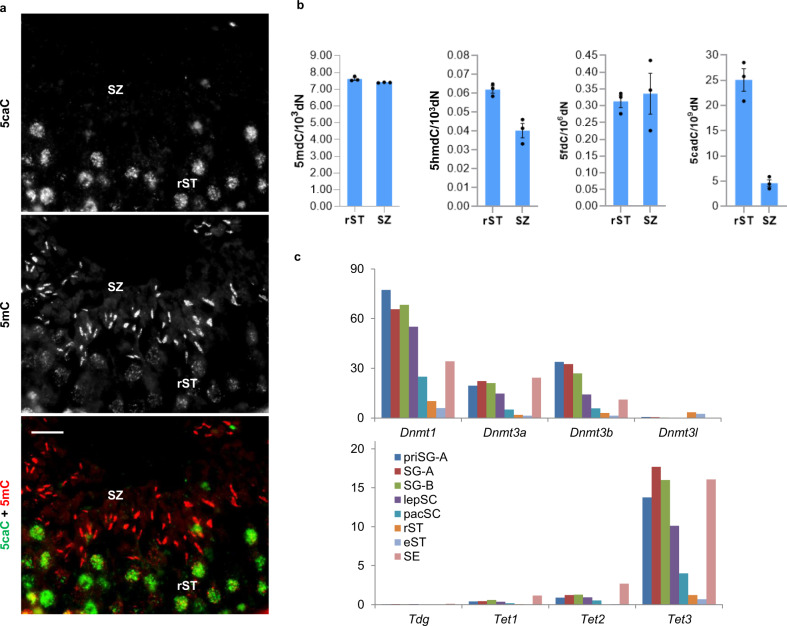
The levels of 5caC substantially decrease in testis germ cells during spermatid maturation. **a** Immunostaining of a representative section of adult murine testis for 5caC and 5mC. Individual channels and merged views are shown. Locations of round spermatids (rST) and spermatozoa (SZ) in the section are indicated. Scale bar is 20 μm. **b** MS quantification of the indicated modified nucleosides in genomic DNA of rST and SZ. *n* = 3 independent MS measurements. Experimental error is expressed as ±SD. **c** Expression of DNA methyltransferases, *TET1/2/3,* and *TDG* mRNAs in the indicated testis cell types according to previously published data set^18,19^ (GSE35005). PriSG-A primitive spermatogonia type A, SG-A spermatogonia type A, SG-B spermatogonia type B, lepSC leptotene spermatocytes, pacSC, pachytene spermatocytes, eST elongated spermatids, rST round spermatids, SE Sertoli cells, SC spermatocytes. The experiments shown in **a** were repeated independently six times with similar results.

To determine the levels of oxidized forms of 5mC in testicular cells, we initially used a method of sensitive immunostaining we previously employed for the detection of 5hmC and 5caC^14,15^. Whereas both germ and somatic cells of the murine testis displayed strong 5mC staining, 5caC was detectable only in germ cells, exhibiting particularly high levels of the signal in rST (Fig. 1a and Supplementary Fig. 1a). We next confirmed the specificity of 5caC staining in competition experiments on testis tissue sections (Supplementary Fig. 1b). Correspondingly, despite obvious detection of 5hmC, we did not observe a 5caC signal in the testes of two mouse models devoid of germ cells: busulfan treated^16^ (busulfan destroys spermatogenic stem cells leading to a lack of any type of germ cells); and adult *GILZ* knockout^17^ mice (where seminiferous tubules also contain only somatic cells) (Supplementary Fig. 1c). Moreover, the 5caC staining was not detectable in the testes of P4 (postnatal day) mice, lacking germ cells of any type other than spermatogonia, but was obvious in spermatocytes at P14 (Supplementary Fig. 1d). Remarkably, although 5hmC and 5mC signals were relatively high in both rST and SZ, the 5caC signal intensity dropped markedly between these two stages (Fig. 1a and Supplementary Fig. 1e, f). In addition, the patterns of nuclear distribution of 5hmC and 5caC were not identical in rST, suggesting that the generation of these two marks is regulated independently in these cells (Supplementary Fig. 1g, h).

To test if the dynamics of 5caC during spermatid maturation are associated with global DNA demethylation, we performed mass spectrometry (MS) detection of 5mC, 5hmC, 5fC, and 5caC in rST, and SZ (Fig. 1b and Supplementary Fig. 2). Although in agreement with a previous study^18^, we could not detect any substantial differences in 5mC content between these stages of spermatogenesis, its oxidized derivatives exhibited different dynamics in our MS experiments (Fig. 1b). Indeed, a 30 percent reduction in the levels of 5hmC was accompanied by a marked 5-fold decrease in 5caC content between rST and SZ, although no changes were detected in 5fC (Fig. 1b), at least under our experimental conditions. Intriguingly, although the MS results confirmed our immunostaining data, the analyses of publicly available RNA expression datasets^18–24^ revealed that contrasting with *Dnmt1/3a/3b/L* and *Tet1/2/3* transcripts, *Tdg* mRNA was not expressed at any appreciable levels in the analyzed testis cell types (Fig. 1c and Supplementary Fig. 3), suggesting that TDG/BER-dependent demethylation is not responsible for the elimination of 5caC during spermatid maturation.

### The patterns of 5caC genomic distribution are highly dynamic during spermiogenesis

Since 5caC exhibited specific dynamics during spermatid maturation, we next profiled the genomic distributions of 5caC, 5mC, and 5hmC (referred to together as mod-Cs) in purified rST (Supplementary Fig. 4) and SZ using DNA immunoprecipitation (DIP) coupled with high-throughput DNA sequencing^25^. After mapping reads to the mouse genome (Supplementary Fig. 5a), we found that the distribution of the densities of mod-Cs across CpG islands, transcription start sites, and most of the regulatory sequences previously identified in mouse testis^26,27^, followed the same pattern in rST and SZ (Supplementary Fig. 5b, c).

**Fig. 2:**
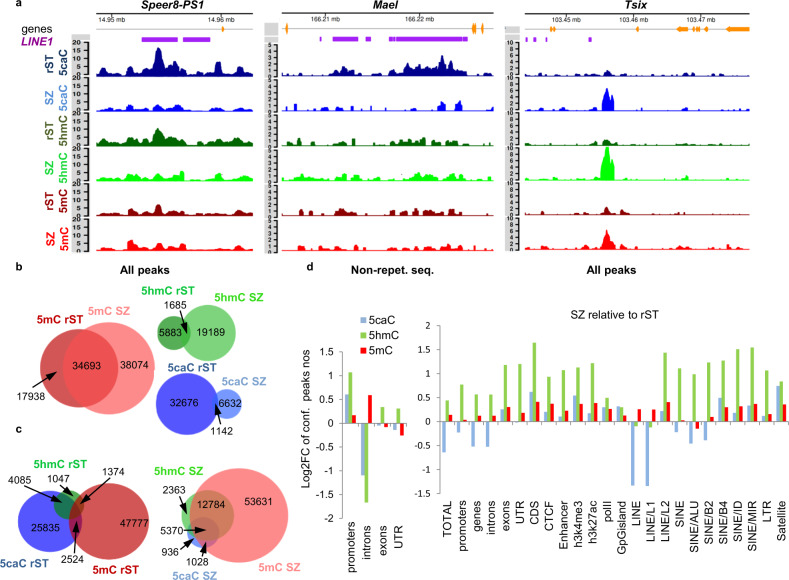
The patterns of 5caC genomic distribution are highly dynamic during spermiogenesis. **a** The coverage plots of mod-Cs densities (CPK) in the introns of spermatogenesis associated glutamate (E)-rich protein 8 (*Speer8-PS1*), maelstrom spermatogenic transposon silencer (*Mael*) and *Tsix* genes exhibiting the differential distribution of 5caC peaks between rST and SZ. **b**, **c** Base pair-specific Venn diagrams showing dynamic changes in the distribution of all confident mod-Cs peaks between rST and SZ stages (**a**) and overlaps between different mod-Cs’ peaks in rST and SZ (**b**). Each circle’s area is equivalent to the number of bases occupied by corresponding peaks in genome sequence space. The numbers of peaks in each category are also indicated. **d** Change of enrichment of mod-Cs peaks localized in non-repetitive sequences and of all mod-Cs peaks in SZ relative to rST at various genomic features.

**Fig. 3:**
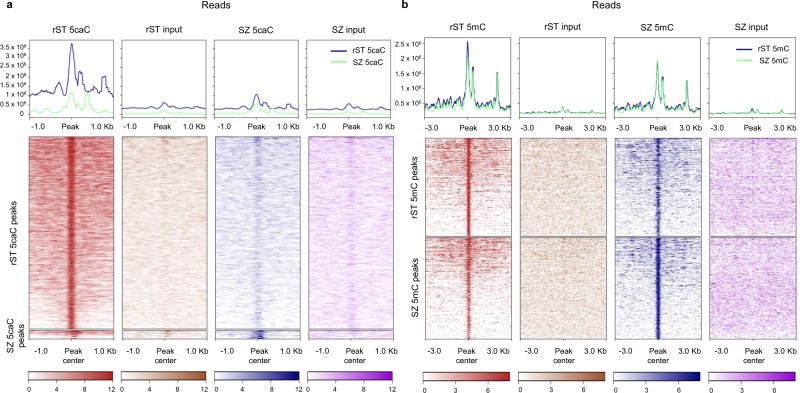
5caC reads density markedly decreased over the majority of the 5caC peaks between rST and SZ stages. **a**, **b** Deeptools heatmaps comparing computed read densities across 5caC (**a**) and 5mC (**b**) SZ and rST peaks (median centered ± 3KB).

To determine the genomic regions enriched in mod-Cs, we performed calling of highly methylated (modified) regions (peaks)^28^, followed by identification of the confident peaks for each sample by comparing the corresponding replicates (Fig. 2a–d and Supplementary Fig. 6a–e, Supplementary Data 1). Consistent with our MS and immunostaining results, the distribution of 5caC confident peaks in rST and SZ was highly dynamic and differed for peaks localized in repetitive and non-repetitive sequences, respectively (Fig. 2b, c and Supplementary Fig. 6a, b). Although the sequence-space occupied by 5caC peaks in non-repetitive sequences increased between rST and SZ (Supplementary Fig. 6a), the majority of the 5caC peaks associated with Transposable Elements (TEs) in rST were not identified in SZ (Fig. 2b). Moreover, while most of the mod-C peaks overlapped with each other in SZ, a large proportion of the 5caC peaks coincided neither with 5mC nor 5hmC peaks in rST (Fig. 2c). Importantly, the majority of 5caC rST peaks did not correspond to 5mC or 5hmC enriched regions in SZ, suggesting that elimination of 5caC from DNA leads to the generation of unmodified cytosine during the rST to SZ transition (Supplementary Fig. 6c). These analyses also revealed that the majority of the 5caC peaks were associated with introns and Long INterspersed Element class-1 (LINE-1 or L1) retrotransposons at the rST stage, but were distributed more evenly between different gene features and classes of repetitive sequences in SZ (Supplementary Fig. 6d, e). Notably, during spermiogenesis, the numbers of 5caC peaks dropped in introns, LINE-1 retrotransposons, and in two classes of Short INterspersed Elements (SINEs) (B1 or Alu-like and B2 elements, Fig. 2d). Importantly, in agreement with our immunostaining and MS-based results (Fig. 1), general 5caC reads density markedly decreased over the majority of the 5caC peaks between rST and SZ stages, whereas the density of 5mC reads did not considerably change at the 5mC peaks (Fig. 3a, b). In summary, these data demonstrated that 5caC patterns are dynamic during spermiogenesis, and that this dynamism is specific for particular classes of genomic sequences.

### 5caC-enriched regions are eliminated from LINE-1 retrotransposons and LINE-1-associated spermiogenesis-specific genes and retained at developmental genes during spermatid maturation

**Fig. 4:**
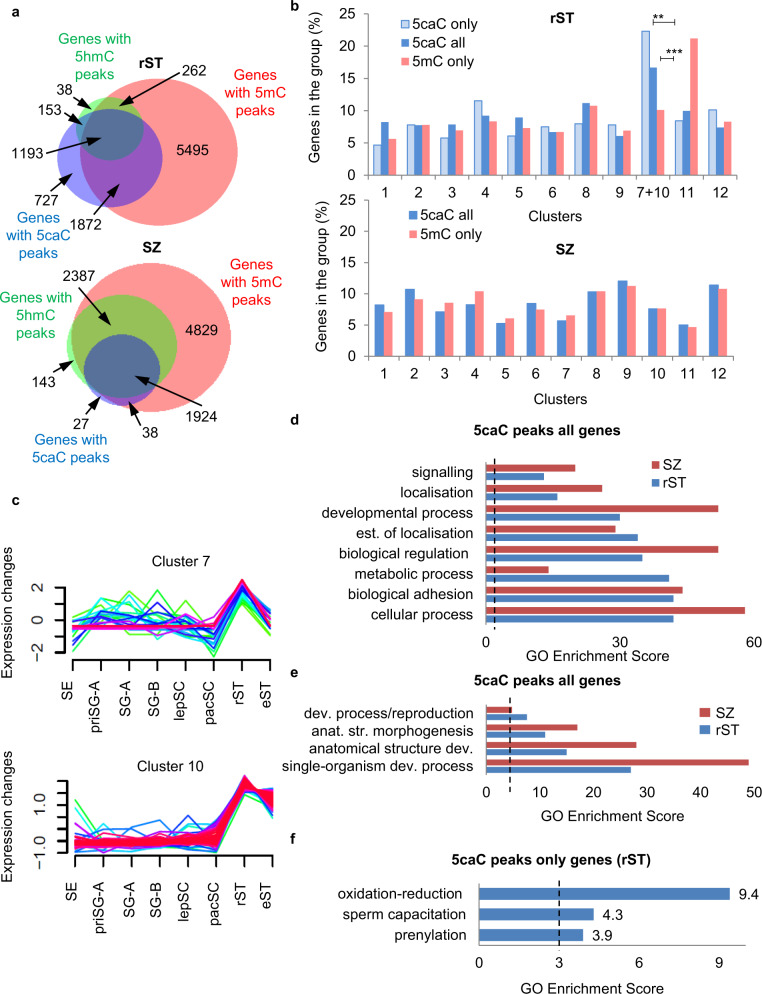
5caC-enriched regions are eliminated from spermiogenesis-specific genes and are retained at developmental genes during spermatid maturation. **a** Venn diagrams showing overlaps between the genes containing mod-Cs peaks in rST and SZ. Each circle’s area is equivalent to the number of genes in each category (indicated). **b** Distribution of all the genes containing 5caC peaks (5caC all), genes encompassing only 5caC but not 5hmC/5mC peaks (5caC only), and genes containing only 5mC but not 5hmC/5caC peaks (5mC only) in rST and SZ regarding to different clusters of gene expression. The significance was determined by *Z*-test of proportions, **Z score = 7.5481 and ***Z score = 9.03 designate significance at 99% confidence interval. **c** The clusters of gene expression enriched in 5caC peak-containing genes in rST. **d** Gene ontology (GO) categories significantly enriched in 5caC peak-containing genes in rST and SZ classified according to their GO score. **e** GO categories associated with developmental processes significantly enriched in 5caC peak-containing genes at rST and SZ stages classified according to their GO score. **f** GO categories enriched in the genes containing exclusively 5caC but not 5hmC/5mC peaks. The significance threshold (GO score = 3) is indicated with a dashed line in **d**–**f**.

To gain insight into the biological significance of the observed dynamics of mod-Cs, we next identified genes containing peaks of mod-Cs in rST or SZ (Supplementary Data 1). Notably, the majority of 5caC and 5hmC peak-containing genes (4311 and 1962, respectively) also displayed 5mC peaks in SZ, contrasting with a substantial number of genes (*n* = 727) containing only 5caC but not 5hmC or 5mC peaks at the rST stage (Fig. 4a). Using previously published datasets^18,19^, we next performed clustering analysis of the genes containing 5caC peaks, genes encompassing only 5caC but not 5hmC/5mC peaks, and genes containing only 5mC but not 5hmC/5caC peaks, according to their expression in different testis cell types (Supplementary Fig. 6f). Although we could not detect any significant differences in the representation of the various clusters between 5caC peak-containing and 5mC only peak-containing genes in SZ, the genes with spermiogenesis-specific patterns of expression (clusters 7 and 10) were significantly enriched in the 5caC peak-containing than the 5mC only peak-containing category in rST (Fig. 4b, c). In contrast, genes transcriptionally silent during spermatid maturation but expressed at earlier stages of spermatogenesis (Supplementary Fig. 6f, cluster 11), were enriched among 5mC only peak-containing genes in rST (Fig. 4b). Subsequent gene ontology (GO) analysis demonstrated that genes associated with “developmental process,” “biological regulation” and “cellular process” categories acquired 5caC peaks between rST and SZ (Fig. 4d). Moreover, we observed retention of 5caC peaks in genes associated with morphogenesis of various anatomical structures, but not with reproduction-linked developmental processes during the rST/SZ transition (Fig. 4e). It is important to note that due to extremely low absolute levels of 5caC in sperm DNA (Figs. 1e and 3a), these 5caC peaks are unlikely to reflect the accumulation of this modification at the corresponding loci and more probably indicate that 5caC is retained at developmental genes in SZ. Interestingly, the genes containing only 5caC peaks in rST were enriched in “sperm-capacitation” and “oxidation reduction” GO categories, reportedly important for spermiogenesis^29^ (Fig. 4f).

**Fig. 5:**
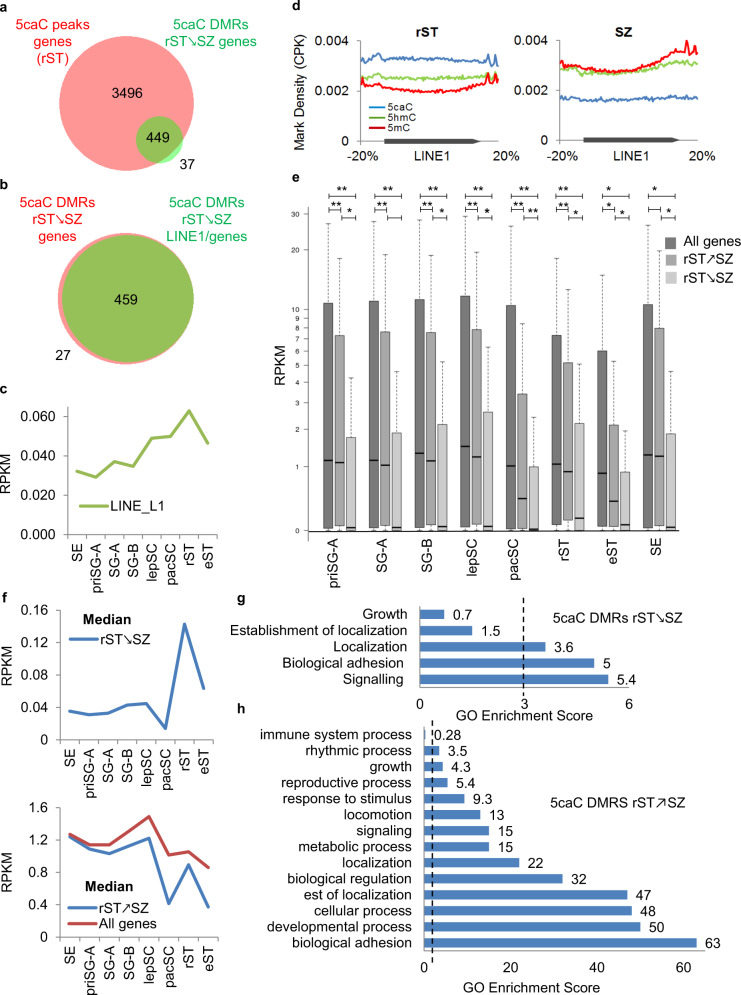
Analysis of the genes containing rST/SZ 5caC DMRs. **a**, **b** Venn diagrams showing overlaps of all genes containing rST↘SZ 5caC DMRs with genes containing 5caC peaks in rST (**a**) or with rST↘SZ 5caC DMRs localized in LINE-1 elements (**b**). Each circle’s area is equivalent to the number of genes in each category (indicated). **c** Expression of LINE-1 retrotransposons in testis cell types (cumulative RPKM values for all referenced LINE-1 elements are shown). **d** mod-Cs densities across LINE-1 elements in rST and SZ. **e**, **f** Box plot of the expression values (**e**) and the median expression values (**f**) of all refseq genes (all genes) and genes containing 5caC rST↗SZ or rST↘SZ DMRs in testis cell types. The elements of the box plots shown in **e** are center line, median; box limits, upper and lower quartiles; whiskers, minimum and maximum of all the data. The significance was determined by a one-way ANOVA test, **p < 0.01; *p < 0.05. **g**, **h** GO categories significantly enriched amongst the genes containing rST↘SZ (**g**) or rST↗SZ (**h**) 5caC DMRs. The significance threshold (GO score = 3) is indicated with a dashed line. Different mod-Cs are plotted together due to space limitations and not for comparison of their absolute values in **d**.

To validate our results by an independent method, we identified differentially methylated regions (DMRs)^30^ at the rST/SZ transition (Supplementary Data 2). We detected 12,539 DMRs that “gain” (rST↗SZ) and 2,874 DMRs that “loose” (rST↘SZ) 5caC in SZ cells (Supplementary Data 2). Importantly, as the density of 5caC reads was substantially decreasing between rST and SZ (Fig. 3a, b), the 5caC enrichment at most of the DMRs “gaining 5caC” at SZ was likely extremely low. Next, we identified genes associated with both classes of 5caC DMRs (Supplementary Data 2). Notably, the majority of the rST↘SZ 5caC DMR-containing genes encompassed LINE-1s in their gene bodies or promoter regions, and corresponded to genes containing 5caC peaks in rST (Fig. 5a, b). In line with this, the 5caC density considerably dropped across the bodies of LINE-1 elements between rST and SZ stages at the same time (Fig. 5c). Moreover, similar to the cluster of expression enriched in 5caC only peak-containing genes (Fig. 5c), the expression of both LINE-1s and rST↘SZ 5caC DMR-containing genes reaches a maximum at the rST stage during spermatogenesis (Fig. 5d–f). The genes encompassing rST↘SZ 5caC DMRs were enriched in “signaling” and “biological adhesion” GO categories and included well-characterized spermiogenesis-specific genes located on the Y-chromosome such as *Ssty2* and *Sly*^31^, which both contain several LINE-1 copies^32^ (Fig. 5g). In contrast, confirming our peak analysis-based results (Fig. 4d), rST↗SZ 5caC DMR-containing genes were associated with a wide range of “developmental process”-related GO categories (Fig. 5h). Thus, using both peak-based and DMR-based approaches, we identified an association of 5caC with LINE-1s and LINE-1-containing spermiogenesis genes in rST and with developmental genes in SZ.

### Transcriptionally active and evolutionary young LINE-1 elements are enriched in 5caC in round spermatids

**Fig. 6:**
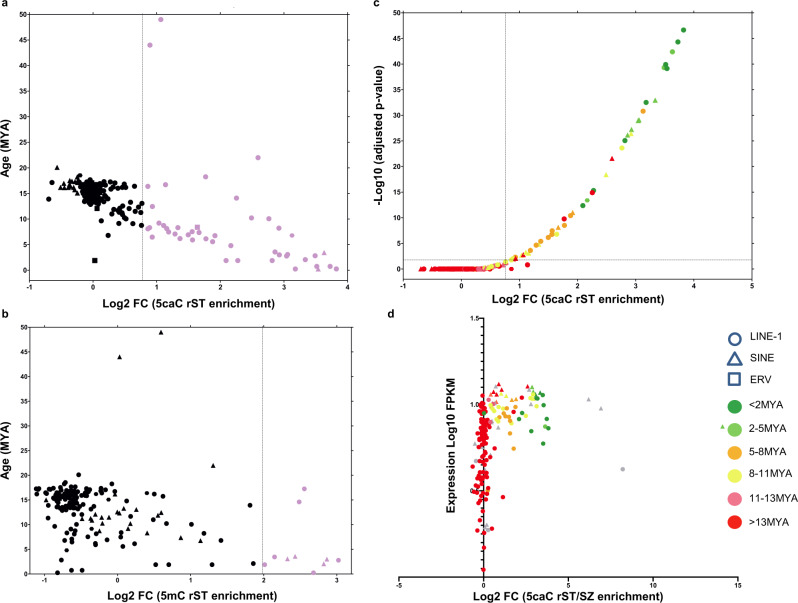
5caC enrichment is associated with actively transcribed and evolutionarily young LINE-1s in round spermatids. **a**, **b** Graphs showing the distribution of different classes of murine TEs depending on their age (million years ago, myo) and the degree of 5caC- (**a**) or 5mC (**b**) enrichment (fold change). Note that only TEs significantly enriched in DIP-seq datasets were included in the analysis. **c** Plot showing the distribution of different classes of murine LINE-1 and SINE elements according to fold change of their 5caC enrichment in rST and its significance (adjusted *p*-value). Elements of different ages are color-coded as shown in **d**. Plot showing the distribution of different classes of LINE-1s and SINEs according to the levels of their transcription in rST (FPKM, Fragments Per Kilobase of transcript) and fold change of their 5caC enrichment at rST/SZ transition. Color- and shape-coding of different elements is shown.

Since we identified an association of 5caC with LINE-1 elements during spermiogenesis, we next enquired how this modification is associated with the evolutionary age and transcriptional activity of these retrotransposons. Several TE types continue to impact the mouse genome, including SINE, LINE-1, and Endogenous Retrovirus (ERV) retrotransposons (reviewed in ref. ^33^). Active LINE-1 retrotransposons replicate in genomes using a copy-and-paste mechanism and have amplified to astonishing numbers in the mouse genome, comprising at least 20% of its genomic mass^34^. Although different LINE-1 subfamilies can be identified within the mouse genome^34^, only three evolutionary young subfamilies of these retrotransposons continue to impact the murine genome: L1Md_G_f_^35^, L1Md_T_f_^36^, and L1Md_A-type^37^ LINE-1s (reviewed in ref. ^29^). Based on two previous studies^38,39^, we categorized murine TEs according to their evolutionary age and determined the degree of 5caC and 5mC enrichment for each of the obtained TE classes. This analysis revealed that the levels of 5caC enrichment of LINE-1 and SINE elements in rST showed a strong inverse correlation with the evolutionary age of these elements (Fig. 6a). In contrast with 5caC, we did not observe a comparable association of 5mC content with the age of TEs (Fig. 6b). Thus, a number of young LINE-1s exhibited moderate or low levels of methylation in round spermatids (Fig. 6b). Importantly, the majority of evolutionary young, originated <2 or 2–5 million years ago (MYA), LINE-1s displayed very substantial degrees of 5caC enrichment in rST (Fig. 6c) and most of them were highly expressed in these cells, as revealed after exploring previously published RNA-seq data set^18,19^ (Fig. 6d). Conversely, we noticed that currently active LINE-1s were considerably enriched in 5caC and highly expressed in rST (Supplementary Fig. 7). Consistently, lower levels of 5caC enrichment were detected in evolutionarily older LINE-1s regardless of the levels of their transcriptional activity (Fig. 6c, d). Next, we compared the distribution of 5caC-, 5mC-DIP-, and whole-transcriptome sequencing reads in the consensus sequences of currently active mouse LINE-1s, focusing on their ORFs and 5′UTRs (Supplementary Fig. 7). These analyses revealed that the promoter regions (i.e., 5′UTR) of evolutionarily young and active LINE-1 retrotransposons (L1Md_A, L1Md_G_f_, and L1Md_T_f_) are substantially depleted of 5caC compared with the ORFs of these retroelements (Supplementary Fig. 7). Remarkably, we did not observe a similar depletion in 5mC in the 5′UTRs of L1Md_A elements (Supplementary Fig. 7).

In summary, our analyses demonstrated that ORFs of evolutionarily young and transcriptionally active LINE-1s are considerably enriched in 5caC during spermatid maturation.

## Discussion

A number of recent studies suggest that, in addition to their roles as intermediates in active demethylation pathway, both 5fC and 5caC may also function as informative epigenetic marks^40–42^. 5fC has been shown to be associated with specific sets of regulatory genomic sequences^43^, and both 5fC and 5caC have been reported to interact with specific groups of candidate “reader” proteins in MS-based proteomics experiments^40^. Most importantly, compared with relatively small numbers of putative 5hmC-binding proteins, potential 5fC and 5caC readers include rather long lists of transcription factors, chromatin remodelers, and histone-modifying enzymes^40,44^. As our results suggest an association of 5caC with actively transcribed loci and show that, in contrast with those of 5mC and 5hmC, 5caC patterns are extremely dynamic during spermiogenesis, our data, collectively, contribute to the emerging body of experimental evidence suggesting a specific function for 5caC in the regulation of gene expression.

Given that reorganization of paternal patterns of 5caC is undergoing during maturation of spermatids, and that *Tdg* is extremely poorly expressed in the mouse testis, our data support the existence of a TDG-independent mechanism of active demethylation, and are in line with previous studies implying that such a mechanism is operative in pre-implantation embryos^6,7^. Interestingly, although it seems unlikely that DNA glycosylases other than TDG contribute to active demethylation as their knockouts do not appear to affect developmental capacity^45^, an unidentified 5caC-specific decarboxylase activity has been detected in mouse ESCs on the basis of isotope tracing^46^. Furthermore, since mammalian de novo DNA methyltransferases DNMT3A and DNMT3B have been reported to convert 5mC and 5hmC to unmodified cytosines in vitro^47,48^, these enzymes may potentially possess DNA decarboxylase activity in specific chromatin microenvironments as well^49^.

Despite the fact that 5fC/5caC are immunochemically detectable in one-cell embryos, a recent study demonstrated that the paternal germline-specific knockout of all three TET proteins does not affect early embryogenesis^50^, implying either dispensability of the spermatogenesis-specific 5mC oxidation for the embryonic development or existence of the mechanisms compensating the absence of this event. This suggests that paternally inherited 5caC is unlikely involved in epigenetic priming of developmental genes in the early embryo. In this context, it is noteworthy that we did not detect any noteworthy association of 5caC with imprinted loci at rST or SZ stages. Thus, although a number of 5caC peaks were localized in the vicinity of previously characterized imprinting control regions (ICRs) (*Usp29*, *Rasgrf1*, *Trappc9*, *Airn*, *Igf2r*)^51^, none of the 5caC peaks or DMRs coincided with ICRs. Moreover, out of ~106 genes known to be imprinted in mouse, only 6 maternally and 5 paternally expressed genes were associated with 5caC peaks in SZ.

In contrast, a large fraction of the mammalian genome is made up of TE-derived sequences. LINE-1 retrotransposons comprise ~20% of the mouse^52^ and human^53^ genomes, and are actively transcribed during germline and early embryonic development in most mammalian species^54–56^, generating new insertions in these cell types^57,58^. Although the transcriptional activity of LINE-1s has long been regarded as a side effect of chromatin remodeling taking place at these developmental stages, a number of recent studies suggest that activation of these retrotransposons is essential for regulating global chromatin accessibility and activity of their host genes during normal development^59–61^. This implies the existence of a complex interplay between the activity of TEs and host gene expression, and suggests that transcription of retrotransposons needs to be finely tuned in both the germline and the early embryo^61–63^. Given that our analysis demonstrates an association between 5caC and transcriptionally active LINE-1s in rSTs, we speculate that the oxidation of 5mC to 5caC may contribute to the delicate regulation of activity of LINE-1 elements and LINE-1-linked genes in these cells. As both 5fC and 5caC have been shown to decrease the rate and substrate specificity of RNA polymerase II transcription and retard transcript elongation on gene bodies^64,65^, these modifications may directly and specifically reduce the transcription rate of active, evolutionarily young LINE-1s, “correcting” the levels of their activity during spermiogenesis. Indeed, together with the presence of LINE-1-associated 5caC-enriched regions in the introns of genes essential for transposon repression, such as piRNA pathway gene Maelstrom (*Mael*)^66,67^, our data add an additional level of complexity to the potential role of this DNA modification in the regulation of LINE-1 elements. In summary, our results suggest that 5caC may be an integral part of an intricate regulatory network governing the activity of LINE-1 retrotransposons during mammalian development, based on finely adjusting the levels of their transcription to avoid accumulating deleterious mutations in the germline genome over evolution.

## Methods

### Animals

Experiments were performed in compliance with the UK and EU guidelines for the care and use of laboratory animals. Animal procedures were subjected to local ethical review (Comite d’Ethique pour l’Experimentation Animale, Universite Paris Descartes; registration number CEEA34.JC.114.12). C57BL/6 wild type, CD1 wild type and CD1 *GILZ Y/-* adult, 4 and 14 dpp male mice were culled by decapitation or by cervical dislocation following sedation by inhalation of CO_2_. Spermatozoa were collected from the caudal segment of the epididymis.

### Immunohistochemistry and confocal microscopy

Immunohistochemistry and confocal microscopy were performed as described^14^. Anti-5hmC mouse monoclonal (Active Motif, 1:5000 dilution), anti-5hmC rabbit polyclonal (Active Motif, 1:5000 dilution), anti-5mC mouse monoclonal (clone 33D3, Diagenode, 1:200 dilution), anti-5caC rabbit polyclonal (Active Motif, 1:500 dilution), and anti-5fC rabbit polyclonal (Active Motif, 1:500 dilution) primary antibodies were used for immunochemistry. Peroxidase-conjugated anti-rabbit secondary antibody (Dako) and the tyramide signal enhancement system (Perkin Elmer, 1:200 dilution, 2 min of incubation with tyramide) were employed for 5caC and 5hmC (rabbit polyclonal antibody) detection. 5hmC (mouse monoclonal antibody) and 5mC were visualized using 555-conjugated secondary antibody (Alexafluor). Control staining without primary antibody produced no detectable signal. Images (500 nm optical sections) were acquired with a Zeiss LSM 700 AxioObserver confocal microscope using a Plan-Apochromat ×63/1.40 Oil DIC M27 objective and processed using Image J and Adobe Photoshop. 2.5XD signal intensity plots were generated using ZEN Zeiss LSM 700 imaging software as described previously^14^.

### Mass spectrometry

Purification of spermatogenic cells was performed by elutriation as previously described^68^. DNA was isolated according to standard procedures. The 2-dimensional ultra-performance liquid chromatography-tandem mass spectrometry (2D-UPLC–MS/MS) analyses were performed according to the method described in ref. ^69^. Briefly, DNA hydrolysates were spiked with a mixture of internal standards in volumetric ratio 4:1, to concentration of 50 fmols/µL of [D_3_]-5-hmdC, [^13^C_10_, ^15^N_2_]-5-formyl-2′-deoxycytidine (5-fdC), [^13^C_10_, ^15^N_2_]-5-carboxyl-2′-deoxycytidine (5-cadC), and [^15^N_5_]-8-oxodG. Chromatographic separation was performed with a Waters Acquity 2D-UPLC system with photo-diode array detector, for the first-dimension chromatography (used for quantification of unmodified deoxynucleosides (dN) and 5-methyl-2′-deoxycytidine (5-mdC)), and Xevo TQ-S tandem quadrupole mass spectrometer for second-dimension chromatography. At-column-dilution technique was used between the first and second dimension for improving retention at trap/transfer column. The columns used were: a Phenomenex Kinetex C18 column (150 mm × 2.1 mm, 1.7 µm) at the first dimension, a Waters X-select C18 CSH (30 mm × 2.1 mm, 1.7 µm) at the second dimension, and Waters X-select C18 CSH (30 mm × 2.1 mm, 1,7 µm) as trap/transfer column. The chromatographic system operated in heart-cutting mode, indicating that selected parts of effluent from the first dimension were directed to trap/transfer column via 6-port valve switching, which served as “injector” for the second-dimension chromatography system. The flow rate at the first dimension was 0.25 mL/min and the injection volume was 0.5–2 µL. The separation was performed with a gradient elution for 10 min using a mobile phase 0.1% acetate (A) and acetonitrile (B) (1–5% B for 5 min, column washing with 30% acetonitrile, and re-equilibration with 99% A for 3.6 min). Flow rate at the second dimension was 0.35 mL/min The separation was performed with a gradient elution for 10 min using a mobile phase 0.01% acetate (A) and methanol (B) (4–50% B for 4 min, isocratic flow of 50% B for 1.5 min, and re-equilibration with 96% A up to next injection). All samples were analyzed in three to five technical replicates of which technical mean was used for further calculation. Mass spectrometric detection was performed using the Waters Xevo TQ-S tandem quadrupole mass spectrometer, equipped with an electrospray ionization source. Collision-induced dissociation was obtained using argon 6.0 at 3 × 10^−6^ bar pressure as the collision gas. Transition patterns for all the analyzed compounds, as well as specific detector settings, were determined using the MassLynx 4.1 Intelli-Start feature.

### 5mC-, 5hmC, and 5caC-DNA IP (DIP)

5mC-, 5hmC, and 5caC-DNA IP (DIP) was performed as described^14,43^. Spermatogenic cells were purified using FACS sorting^31^. Fractions were assessed under phase optics and the purity of rST fraction was consistently more than 95%. Genomic DNA was isolated from rST and SZ cells or hPSCs according to standard procedures and fragmented to 100–300 bp using Diagenode Bioruptor Standard UCD-200. 10 μg of genomic DNA was used for immunoprecipitation. 5hmC- and 5caC-DIP were carried out using rabbit polyclonal antibodies (Active Motif) and magnetic anti-rabbit Dynabeads (Invitrogen). Mouse monoclonal antibody (clone 33D3, Diagenode) and anti-mouse Dynabeads (Invitrogen) were used for 5mC-DIP. Specificity of the antibodies was assessed in DIP experiments with modified oligonucleotides as described^14^.

### Library preparation and high-throughput sequencing

SOLiD sequencing libraries (rST and SZ samples) were prepared from 5caC-, 5hmC-, and 5mC-DIP enriched DNA as stated in the Lifetech Solid 5500 Chip-Seq library preparation guide. Enzymes and reagents were used from the 5500 SOLiD Fragment Library Core Kit (Life Technologies, 4464412). Fifteen cycles of library amplification were carried out using primers specific to the library sequencing adapters. Barcoded DNA fragment libraries were quantified using the Kapa Library Quantification kit (Kapa Biosystems, KK4823) and pooled equally. The Solid EZ bead system was used according to the manufacturer’s guidance to prepare ePCR and enrichment of templated beads.

### Bioinformatics analysis

5mC, 5hmC, and 5caC-DIP-SOLiD read alignment were performed as follows. The 75 bp color space (cs) SOLiD reads were aligned to the mouse genome (mm10) using the aligner LifeScope (Life Technologies). The alignment parameters were modified to use a seed length of 60 cs with a 6 cs miss-match allowance. Reads that aligned to more than 99 genomic positions were discarded. Reads were mapped with a low miss-match tolerance. The primary alignment position was recorded for each read. If a read mapped to more than one position with the same best alignment score then one of these positions was selected at random as the primary alignment position. Highly modified (methylated) regions (HMRs, peaks) were first identified from alignment BAM files for each replicate sample using the peak-calling software MACS1.4^28^. MACS1.4 parameters were: effective genome size = 1.87e+09, band width = 300, model fold = 10,30, *p*-value cutoff = 1.00e−10. The input sample for the corresponding cell type was used as the background control. A *p*-value of 1e−10 was used to determine peaks. Highly confident peaks were subsequently identified for each sample by comparing the replicate peaks using the bioconductor package DiffBind. DiffBind parameters were: bScaleControl = TRUE, bParallel = TRUE, bCorPlot = TRUE, consensus = -DBA_REPLICATE. Differentially methylated (modified) regions (DMRs) between cell types were determined using the bioconductor package MEDIPS v1.14.0^30^. MEDIPS parameters were: uniq = TRUE, extend = 300, shift = 0, ws = 500. Both replicate sample BAM files and the corresponding input sample BAM file for each cell type were used as input. A *p*-value of 0.01 was used to identify significant DMRs. Each peak or DMR was assigned to a gene if it was located within 1 Kb of the gene coding sequence. The coordinates of gene features and CpG islands for the mouse genome assembly mm10 were sourced from the UCSC Genome Browser refGene set^70^. The coordinates for repeat elements were provided by RepeatMasker open-4.0.3 - Repeat Library 20130422. The coordinates of transcription factors and histone modification sites were sourced from previously published data set^26,27^, extracted from the ENCODE project (http://genome.ucsc.edu/ENCODE/) and converted to the mm10 assembly coordinates using LiftOver^28^. The genomic coordinates of features were compared using BedTools^71^. Pybedtools were used to generate base pair-specific Venn diagrams^72^. The coverage plots were generated with the R package Gviz and BEDtools. For the analysis of gene expression in spermatogenic cell types, the RNA-seq reads from a previously published data set^18,19^ were re-analyzed using the mouse genome assembly mm10. Reads were aligned to the mouse genome using TopHat2^73^. Read counts per gene were subsequently calculated with Htseq-count^74^ and normalized gene expression values (RPKM) calculated. The RPKM values for LINE-1 elements were calculated using primary read alignments. Gene expression profiles were clustered according to changes in expression value across the testis cell types using the bioconductor package Mfuzz^75^. Statistically enriched Gene Ontology (GO) categories were determined within lists of genes using Partek Genomics Suite version 6.6 Gene Set ANOVA. Area-proportional Venn diagrams for lists of genes were generated using BioVenn^76^. The heatmap plots that compare computed read densities across 5caC and 5mC SZ and rST peaks were generated using deeptools2 plotHeatmap tool^77^. The deeptools computeMatrix parameters were: reference-point–referencePoint center -a 3000 -b 3000–skipZeros–missingDataAsZero. To quantify Transposable Elements at the subfamily level, we used SQuIRE (Software for Quantifying Interspersed Repeat Elements)^78^. Briefly, DIP-SOLiD or RNA-seq-Illumina reads from the previous study^18,19^ (GEO accession number GSE35005) were aligned to mm10 reference genome, and the quantification stage was performed using the SQuIRE-specific algorithm, which incorporates both unique and multi-mapped reads, generating output read counts and fragments per kilobase transcript per million reads (FPKM) for each TE locus. TE count tables were used for differential (expression) analysis of genes and TEs using DESeq2 via Squire Call tool. DESeq2^79^ estimates variance-mean dependence in count data, and tests for differential (expression) analysis, based on a model using the negative binomial distribution. The age of TEs was estimated as described in ref. ^34^, and TE-consensus sequences were obtained from DFAM^80^.

### Statistics and reproducibility

At least three independent experiments were carried out for MS and immunostaining experiments. All experiments were replicated independently. DIP was performed in two biologically independent experiments. We observed a generally good correlation between the replicates. Statistical tests used for individual experiments are described in corresponding figure legends. Signal intensity and MS data were plotted and analyzed in GraphPad Prism 7.04.

### Reporting summary

Further information on research design is available in the Nature Research Reporting Summary linked to this article.

## Supplementary information

Supplementary Information

Description of Additional Supplementary Files

Supplementary Data 1

Supplementary Data 2

Supplementary Data 3

Reporting Summary

## Data Availability

The rST and SZ deep sequencing data have been deposited in the EBI’s European Nucleotide Achieve (ENA) (http://www.ebi.ac.uk/ena) under accession number PRJEB8358. MS source data for Fig. 1b can be found in Supplementary Data 3. The confocal raw data and all other data supporting the conclusions of this study are available from the corresponding author upon reasonable request.

## Code availability

The in-house scripts used for the analysis can be found in the following online repository (https://bitbucket.org/thomgiles/adac0175-code).
