# Supplementary material for: LINE-1 transcription in round spermatids is associated with accretion of 5-carboxylcytosine in their open reading frames
Source: Commun Biol. 2021 Jun 7;4:691. doi: 10.1038/s42003-021-02217-8 (PMC8184969; doi:10.1038/s42003-021-02217-8)
Supplement: Supplementary file 1 — Supplementary Information [file 42003_2021_2217_MOESM1_ESM.pdf]

Supplementary Information

Title: LINE-1 transcription in round spermatids is associated with accretion of 5-carboxylcytosine in their open reading frames

Authors: Martin J. Blythe, Ayhan Kocer, Alejandro Rubio-Roldan, Tom Giles, Abdulkadir Abakir, Côme Ialy-Radio, Lee M. Wheldon, Oxana Bereshchenko, Stefano Bruscoli, Alexander Kondrashov, Joël R. Drevet, Richard D. Emes, Andrew D. Johnson, John R. McCarrey, Daniel Gackowski, Ryszard Olinski, Julie Cocquet, Jose L. Garcia-Perez and Alexey Ruzov

Supplementary Figures

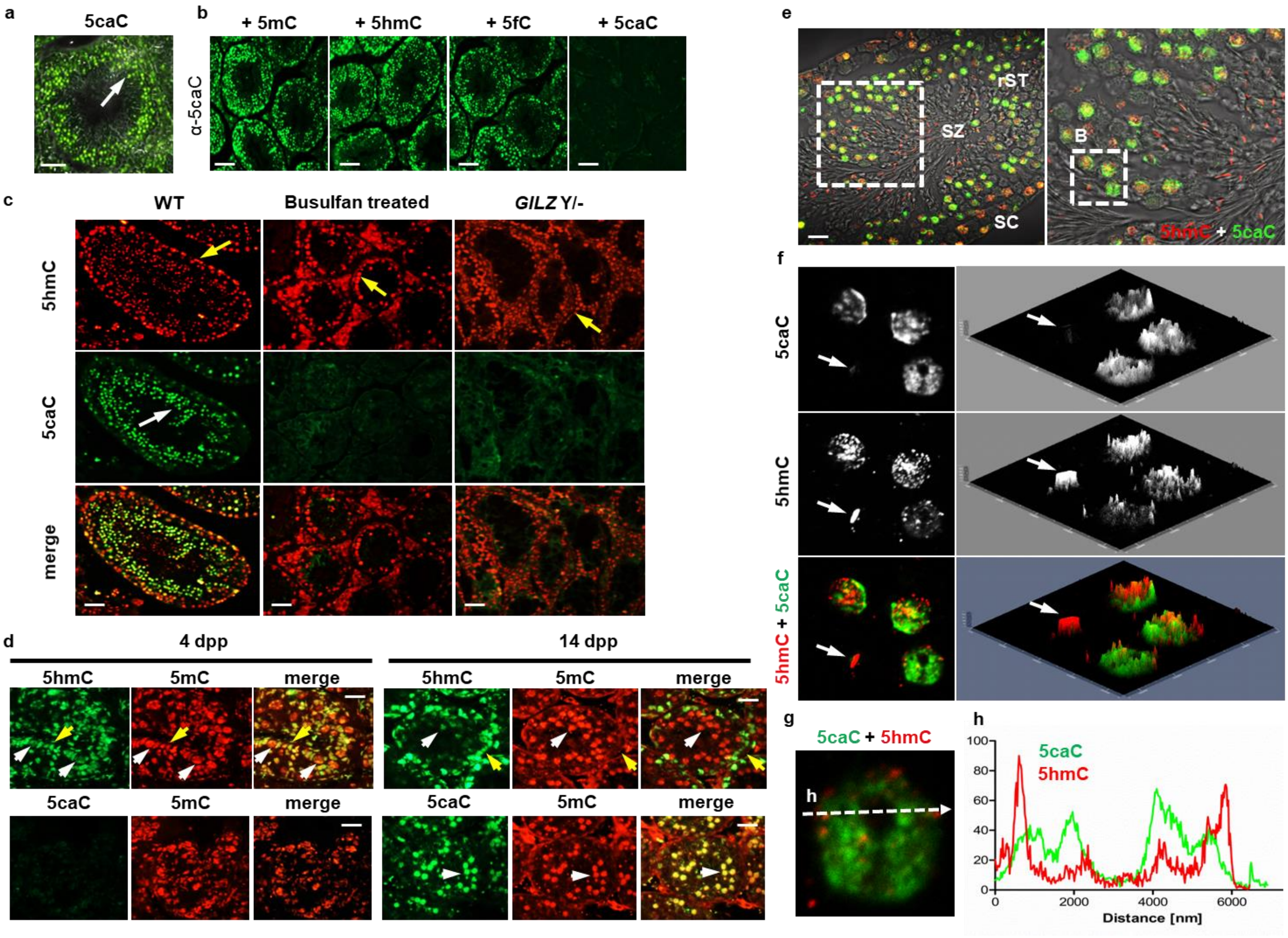

Supplementary Figure 1. 5fC/5caC are detectable in testis germ cells but not in somatic testis cell types. (**a**) 5caC immunostaining in a representative section of mouse adult testis. 5caC channel overlapped with phase image is shown. Testis germ cells are arrowed. (**b**) Immunostaining of serial murine testis sections using anti-5caC antibody in the presence of 5mC-, 5hmC-, 5fC- and 5caC-enriched DNA (indicated). Single channels for 5caC are shown. 5caC staining disappears in the presence of 5caC- but not 5mC-, 5hmC or 5fC-enriched DNA. (**c**) Immunostaining of representative sections of wild type (WT), busulfan treated and *GILZ* Y/- testes using anti-5caC and anti-5hmC antibodies. Single channels and merge views are shown. Testis germ cells are indicated with white and somatic cells with yellow arrows. (**d**) Immunostaining of representative sections of P4 and P14 testes for 5hmC and 5mC (upper panels) or 5caC and 5mC (lower panels). Individual channels and merged views are shown. Testis germ cells (spermatogonia in P4, and spermatocytes in P14) are indicated with white and somatic cells with yellow arrows. (**e**) Co-detection of 5caC with 5hmC in a representative section of wild type testis imaged at two magnifications. Merge views are shown. (**f**) 5caC and 5hmC signals in the nuclei of round spermatids (rST) and spermatozoa (SZ) in the region of testis section marked with dotted rectangle in (**e**). Single channels, merged views and 2.5XD intensity plots are presented. SZ nucleus is arrowed. (**g, h**) 5caC and 5hmC immunostaining in a representative rST nucleus (**g**) and signal intensity profile across its region marked with dotted arrow (**h**). Scale bars are 100 µm in (**a-c**) and 20 µm in (**d, e**). The experiments shown in (**a, b, e-h**) and in (**c, d**) were repeated independently 4 and 3 times correspondingly with similar results.

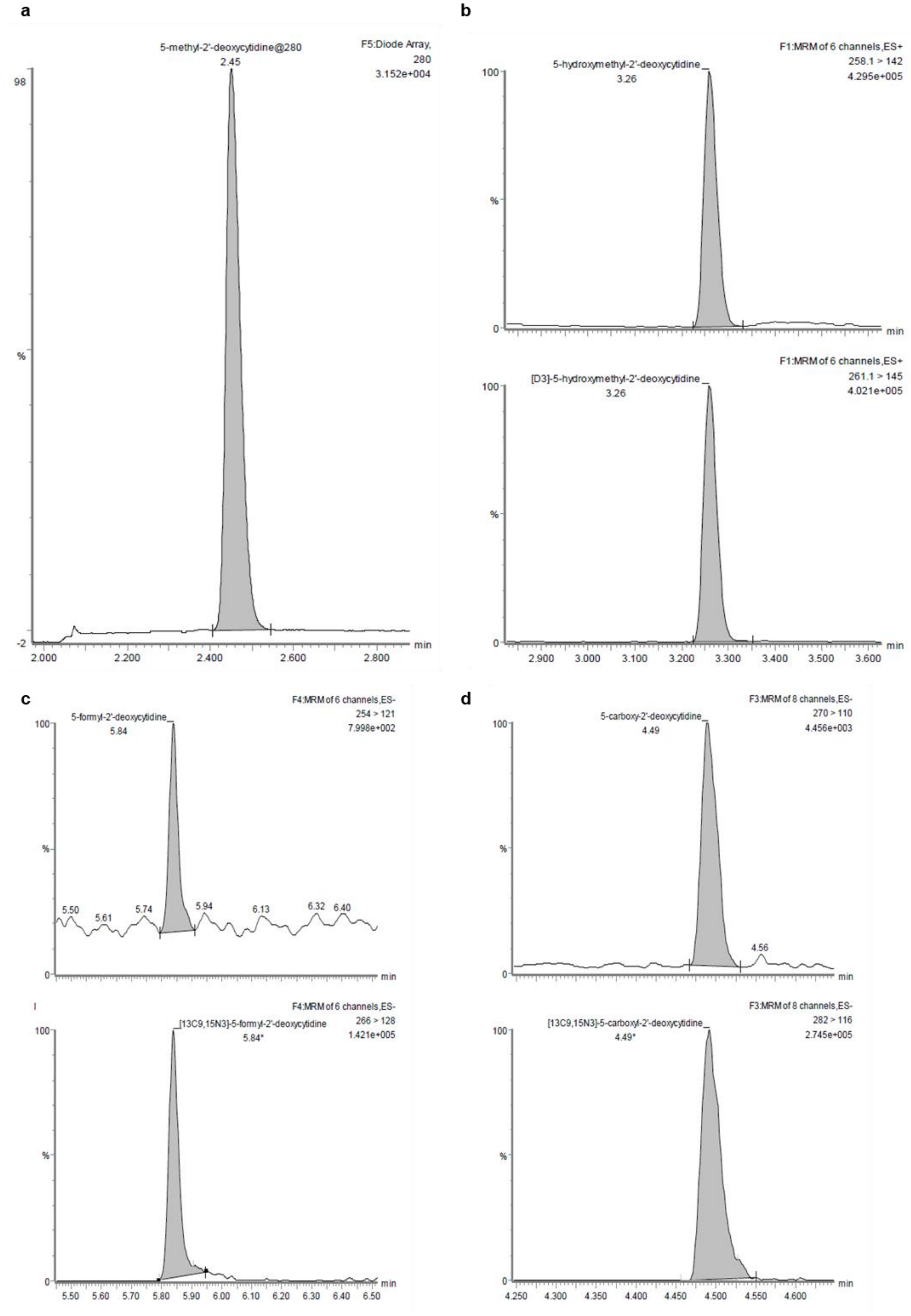

Supplementary Figure 2. Representative UPLC-UV-MS/MS chromatograms of 2 µL of hydrolyzed genomic DNA of round spermatid, spiked with stable-isotope labelled internal standards. (**a**) 5-methyl-2'-deoxycytidine (UV trace at 280 nm), (**b**) 5-(hydroxymethyl)-2'-deoxycytidine, (**c**) 5-formyl-2'-deoxycytidine, (**d**) 5-carboxy-2'-deoxycytidine.

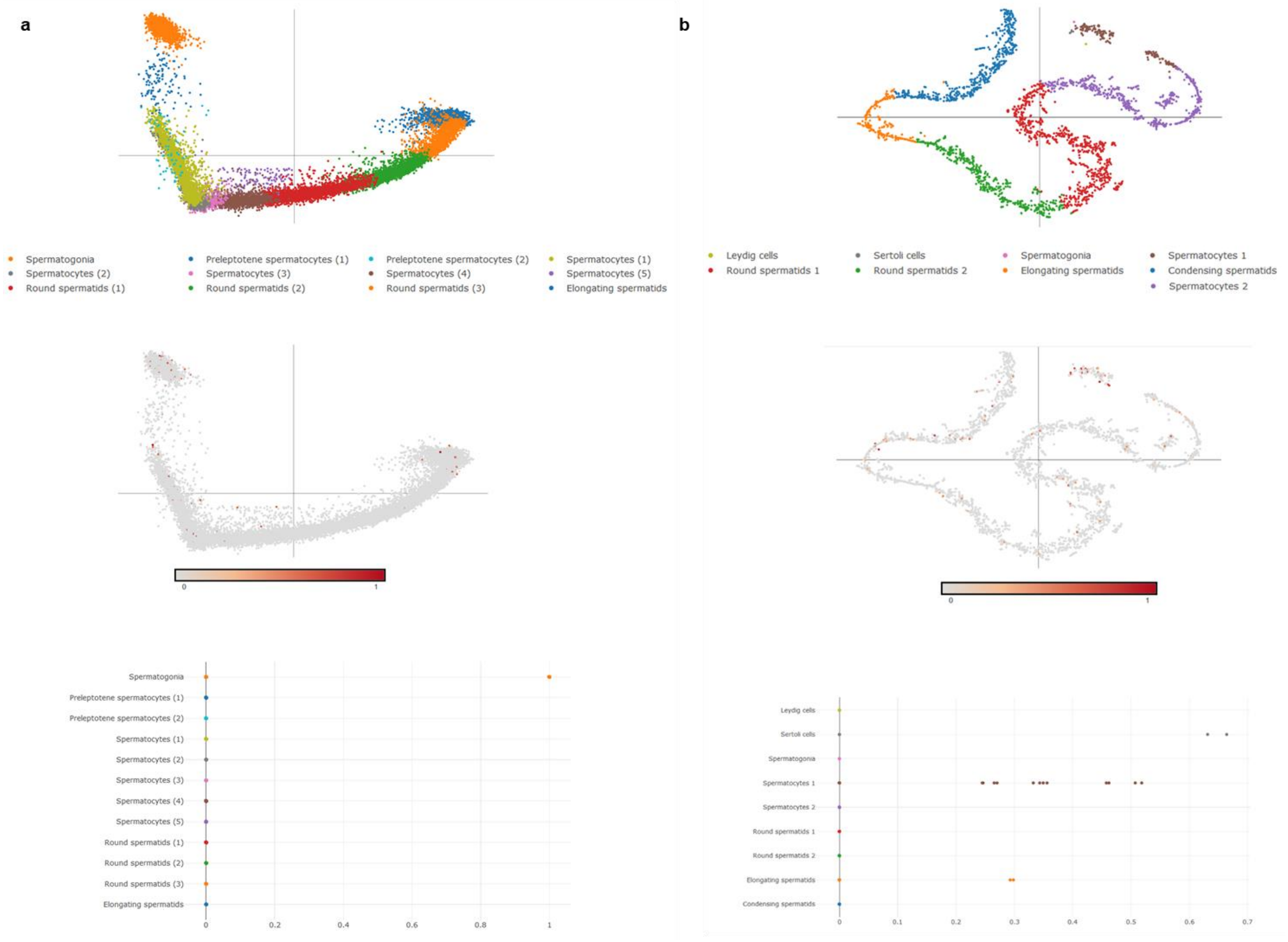

Supplementary Figure 3. Spermatogenetic cells exhibit low level of *Tdg* mRNA. Scatter plots generated by ReproGenomics Viewer website (http://rgv.genouest.org)[20] showing expression of *Tdg* transcript in mouse male germ cells according to single cell RNA-Seq datasets obtained from references 21, 22 (**a**) and 23, 24 (**b**).

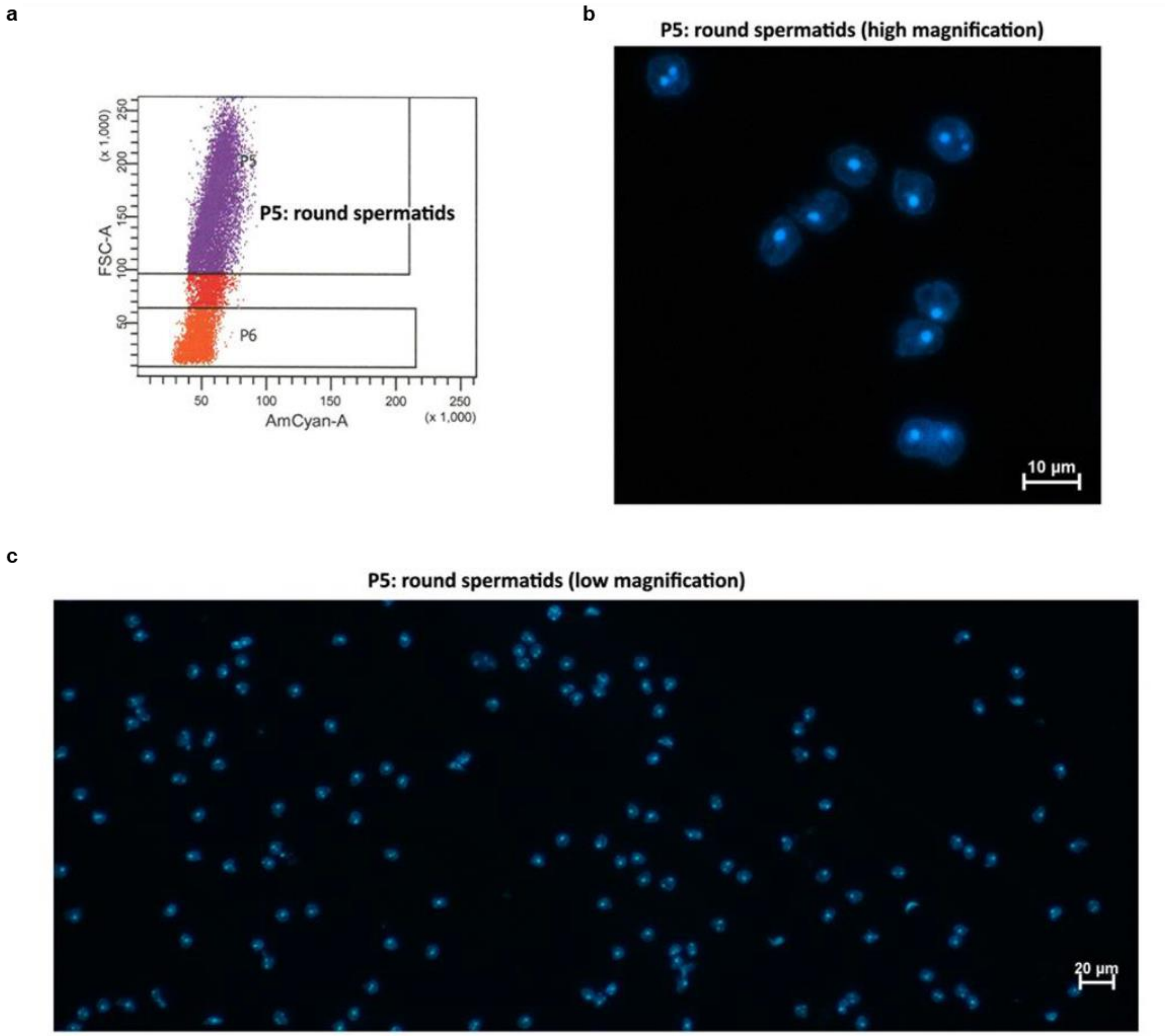

Supplementary Figure 4. Purity assessment of the rST cell population used for mod-Cs DIP experiments. (**a**) The graph represents a typical scatter plot obtained after FACS, used to discriminate between round spermatids (P5, used in the present study) and elongating/condensing spermatids (P6) according to the Forward Scatter Parameters. (**b, c**) Two pictures of the corresponding P5 cells are shown (taken at low and high magnification). DAPI (in blue) was used to stain the nuclei and identify round spermatids. Approximately 95% of P5 cells are round spermatids.

a

Sample	Reads	Mapped Reads	Total Mapping Positions	Uniquely Aligned Reads
S_input	46,437,296	33,656,276	78,321,983	29,606,001
SZ_5caC_1	28,582,084	2,337,278	21,923,495	1,529,398
SZ_5caC_2	53,010,894	9,065,543	77,044,352	6,246,643
SZ_5hmC_1	30,629,127	7,419,419	66,831,078	4,818,955
SZ_5hmC_2	39,705,182	7,566,915	42,644,216	5,711,283
SZ_5mC_1	36,228,917	10,662,548	131,711,476	6,498,747
SZ_5mC_2	14,282,757	12,670,152	154,137,173	7,679,669
F_input	46,848,209	33,430,573	88,644,267	28,998,097
rST_5caC_1	37,041,036	5,653,685	71,042,916	3,072,343
rST_5caC_2	47,273,391	13,904,857	196,507,038	7,257,179
rST_5hmC_1	36,200,923	8,028,663	100,752,501	4,615,717
rST_5hmC_2	17,387,476	7,988,368	83,336,790	4,978,754
rST_5mC_1	36,642,021	10,782,542	177,555,028	5,113,435
rST_5mC_2	39,610,803	8,761,668	128,674,457	4,684,223

b

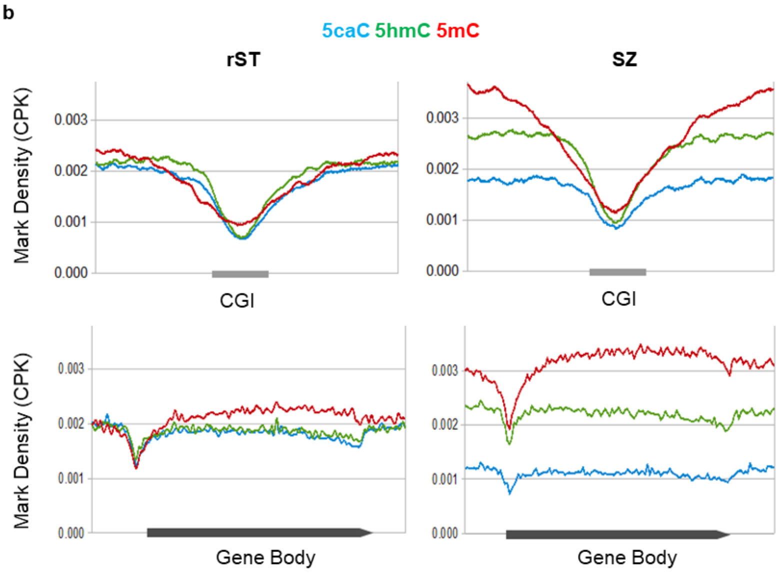

c

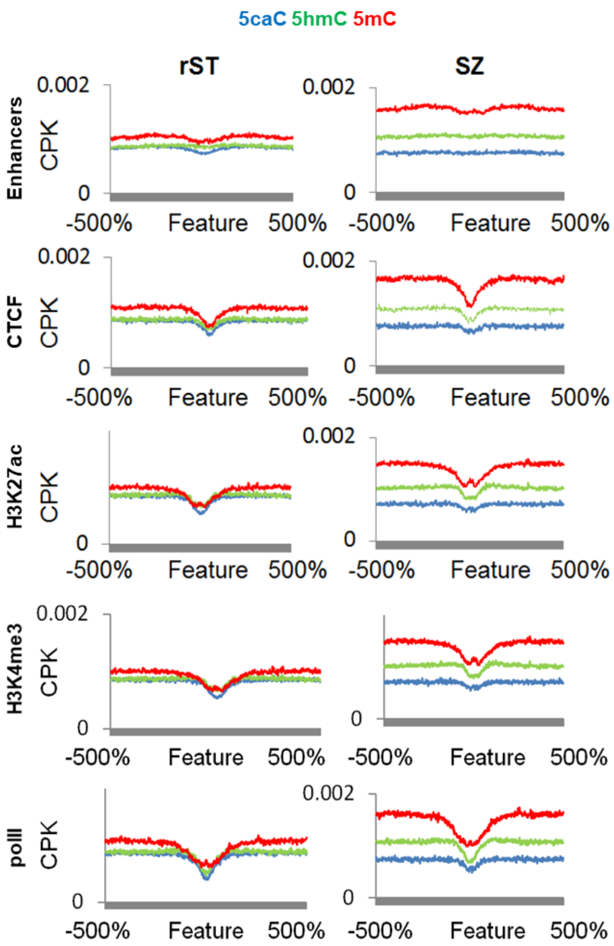

Supplementary Figure 5. Distribution of 5mC, 5hmC and 5caC in various genomic regions in rST and SZ cells. (**a**) Read metrics for 5mC, 5hmC and 5caC-DIP performed on rST and SZ cells. Mapped Reads are total primary read alignments, Total Mapping Positions are multimap alignment positions, and Uniquely Aligned Reads are reads that only align to one genome position. (**b**, **c**) Plots showing distribution of 5mC, 5hmC and 5caC densities across all referenced CpG-islands and all RefSeq genes as well as indicated genomic features (insulators bound by CTCF, H3K27ac and H3K4me3 sites, polII binding sites and enhancers) at rST and SZ stages. The analysis was performed using uniquely aligned reads.

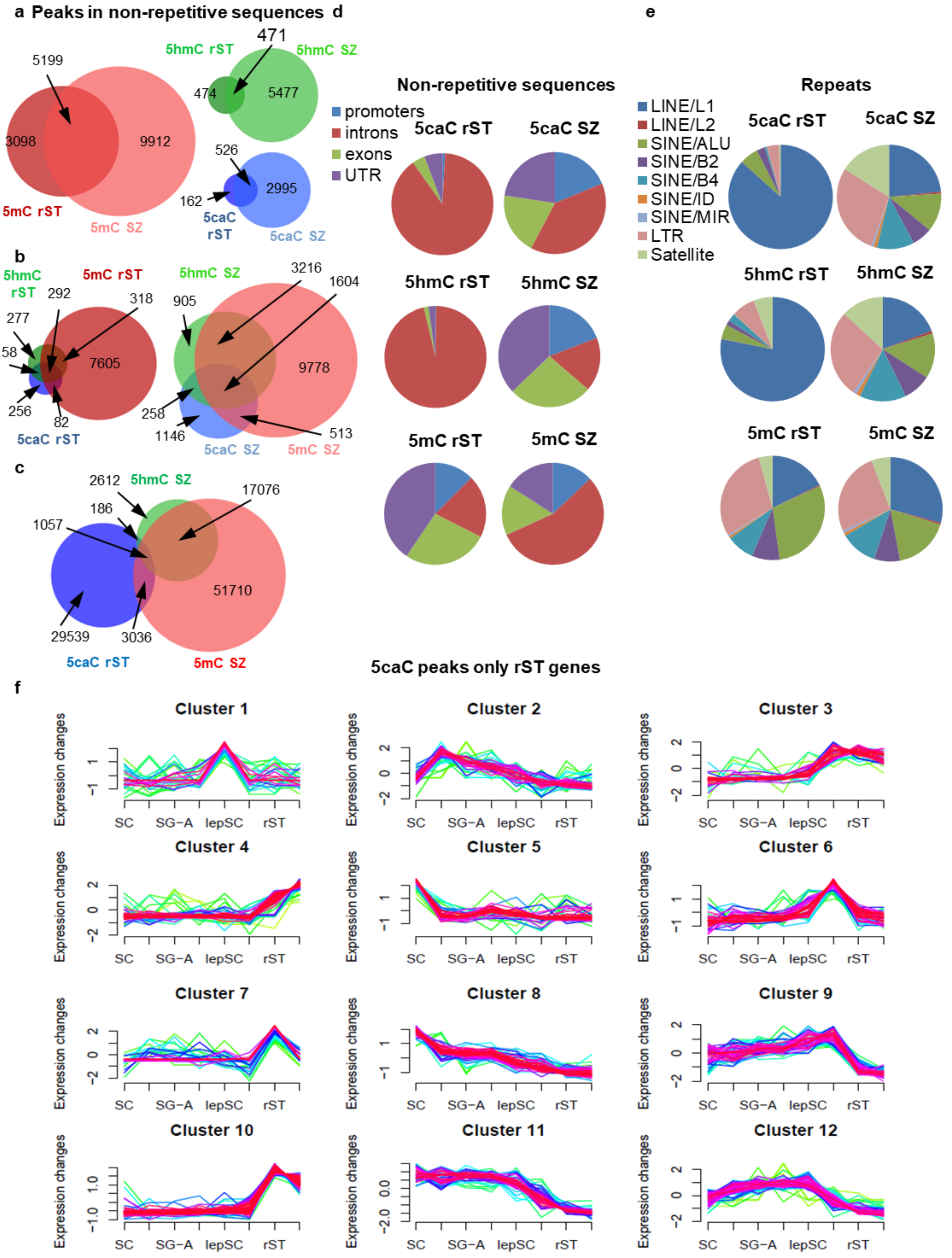
a Peaks in non-repetitive sequences
5199
3098
9912
5mC rST
5mC SZ
471
5hmC rST
5hmC SZ
474
5477
526
2995
162
5caC rST
5caC SZ
b
5hmC rST
5mC rST
5hmC SZ
292
277
318
58
7605
256
82
5caC rST
905
3216
1604
9778
258
1146
513
5caC SZ
5mC SZ
c
2612
5hmC SZ
17076
186
1057
51710
29539
3036
5caC rST
5mC SZ
d
Non-repetitive sequences
promoters
introns
exons
UTR
5caC rST
5caC SZ
5hmC rST
5hmC SZ
5mC rST
5mC SZ
e
Repeats
LINE/L1
LINE/L2
SINE/ALU
SINE/B2
SINE/B4
SINE/ID
SINE/MIR
LTR
Satellite
5caC rST
5caC SZ
5hmC rST
5hmC SZ
5mC rST
5mC SZ
5caC peaks only rST genes
f
Cluster 1
Cluster 2
Cluster 3
Cluster 4
Cluster 5
Cluster 6
Cluster 7
Cluster 8
Cluster 9
Cluster 10
Cluster 11
Cluster 12
Expression changes
SC
SG−A
lepSC
rST

Supplementary Figure 6. Genomic distribution of 5mC, 5hmC and 5caC enriched regions in rST and SZ cells. (**a, b**) Area-proportional Venn diagrams showing dynamic changes in distribution of 5mC, 5hmC and 5caC confident peaks between rST and SZ stages (**a**) presented alongside the Venn diagrams demonstrating overlaps between the 5caC, 5hmC and 5mC peaks at rST and SZ stages (**b**). The results of analysis performed on confident peaks localised exclusively in non-repetitive sequences are shown. (**c**) Area-proportional Venn diagram showing overlap between 5caC peaks in rST and 5hmC/5mC peaks at SZ stage. The results of analysis performed on all confident peaks are shown. Each circle's area is equivalent to the number of bases occupied by corresponding peaks in genome sequence-space and the numbers of peaks in each category are indicated in (**a-c**). (**d, e**) Pie-charts showing composition of 5caC, 5hmC and 5mC enriched regions in non-repetitive sequences (**d**) and repeats (**e**) at SZ and rST stages based on the numbers of confident peaks in each category. (**f**) The results of clustering analysis of the genes containing only 5caC but not 5hmC or 5mC peaks according to their expression in different testis cell types.

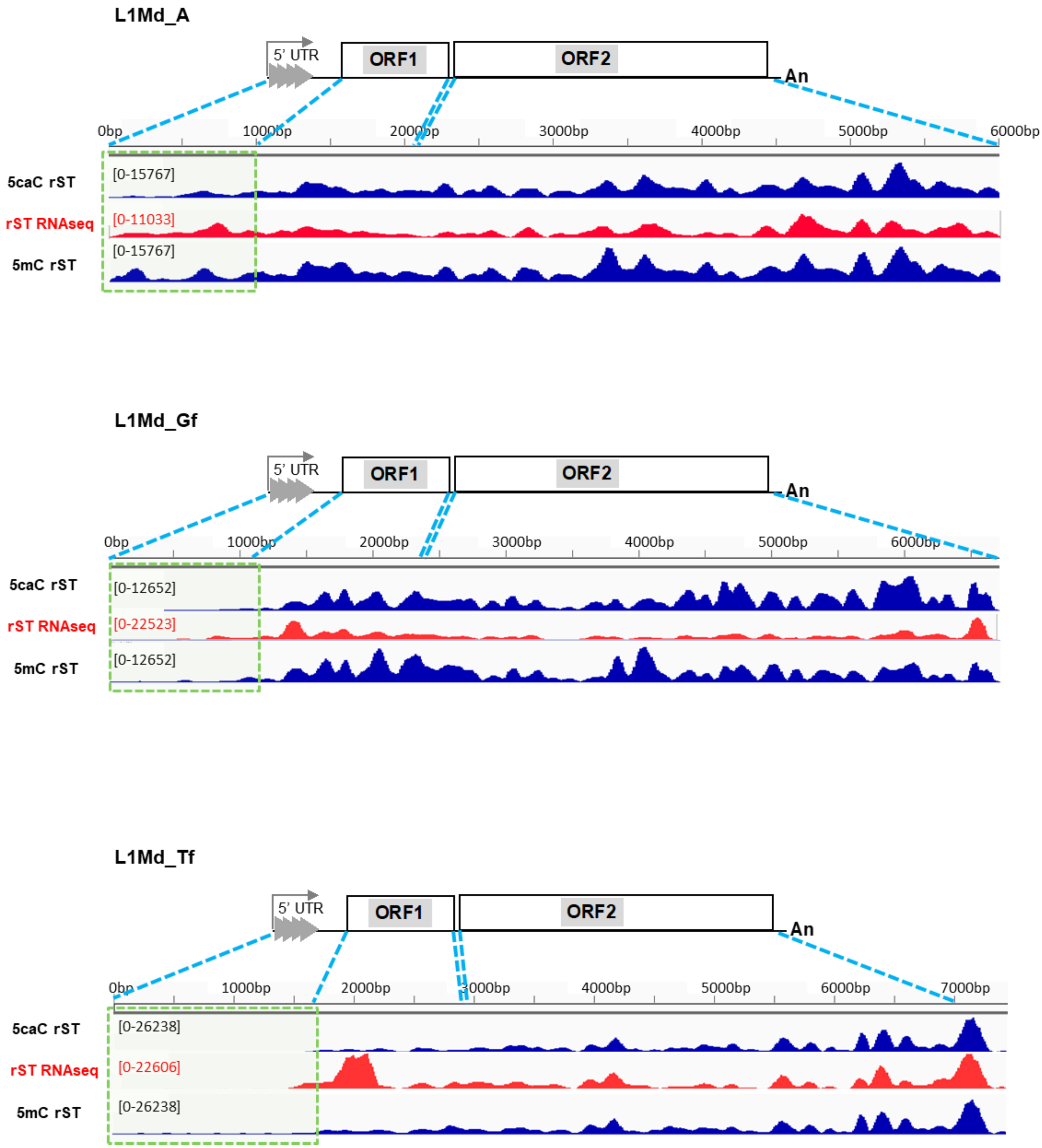

Supplementary Figure 7. 5caC is enriched at the open reading frames of evolutionarily young LINE-1s in round spermatids. Comparison of the distribution of normalized 5caC and 5mC DIP reads together with normalized RNA-seq reads across the consensus sequence of the indicated LINE-1 subfamily member in rST. The LINE-1s promoter regions are marked with green shaded rectangles.
