# Supplementary material for: LINE-1 transcription in round spermatids is associated with accretion of 5-carboxylcytosine in their open reading frames
Source: Commun Biol. 2021 Jun 7;4:691. doi: 10.1038/s42003-021-02217-8 (PMC8184969; doi:10.1038/s42003-021-02217-8)
Supplement: Supplementary file 2 — Description of Additional Supplementary Files [file 42003_2021_2217_MOESM2_ESM.pdf]

Description of Additional Supplementary Files

File name: Supplementary Data 1

Description: 5caC, 5hmC and 5mCenriched regions (peaks) in rST and SZ cells.

File name: Supplementary Data 2

Description: 5caC, 5hmC and 5mC DMRs at rST/SZ transition.

File name: Supplementary Data 3

Description: MS source data for Figure 1b.
