# Supplementary material for: LINE-1 transcription in round spermatids is associated with accretion of 5-carboxylcytosine in their open reading frames
Source: Commun Biol. 2021 Jun 7;4:691. doi: 10.1038/s42003-021-02217-8 (PMC8184969; doi:10.1038/s42003-021-02217-8)
Supplement: Supplementary file 6 — Reporting Summary [file 42003_2021_2217_MOESM6_ESM.pdf]

nature research

Corresponding author(s): Alexey Ruzov, Richard Emes

Last updated by author(s): Apr 27, 2021

Reporting Summary

Nature Research wishes to improve the reproducibility of the work that we publish. This form provides structure for consistency and transparency in reporting. For further information on Nature Research policies, see our Editorial Policies and the Editorial Policy Checklist.

Statistics

For all statistical analyses, confirm that the following items are present in the figure legend, table legend, main text, or Methods section.

n/a	Confirmed	
☐	☒	The exact sample size (*n*) for each experimental group/condition, given as a discrete number and unit of measurement
☐	☒	A statement on whether measurements were taken from distinct samples or whether the same sample was measured repeatedly
☐	☒	The statistical test(s) used AND whether they are one- or two-sided<br>*Only common tests should be described solely by name; describe more complex techniques in the Methods section.*
☐	☒	A description of all covariates tested
☐	☒	A description of any assumptions or corrections, such as tests of normality and adjustment for multiple comparisons
☐	☒	A full description of the statistical parameters including central tendency (e.g. means) or other basic estimates (e.g. regression coefficient) AND variation (e.g. standard deviation) or associated estimates of uncertainty (e.g. confidence intervals)
☐	☒	For null hypothesis testing, the test statistic (e.g. *F*, *t*, *r*) with confidence intervals, effect sizes, degrees of freedom and *P* value noted<br>*Give P values as exact values whenever suitable.*
☒	☐	For Bayesian analysis, information on the choice of priors and Markov chain Monte Carlo settings
☒	☐	For hierarchical and complex designs, identification of the appropriate level for tests and full reporting of outcomes
☐	☒	Estimates of effect sizes (e.g. Cohen's *d*, Pearson's *r*), indicating how they were calculated

Our web collection on statistics for biologists contains articles on many of the points above.

Software and code

Policy information about availability of computer code

Data collection	Confocal images were obtained using Zeiss LSM 710 Zen 2012 Black Edition Service pack 5 imaging software.
Data analysis	Deep sequencing data were processed using open source software tools. The following packages were used: LifeScope (LifeTechnologies), MACS1.4, Samtools version 1.3.1, MEDIPS v1.14.0, LiftOver, Gviz, BEDtools, TopHat2, Htseq-count, Mfuzz, Partek Genomics Suite version 6.6 Gene 483 Set ANOVA, Picard Tools Dedup version 2.5.0, MACS2.1.1, PhantomPeakQualTools version - Feb 13, 2012, Bedtools version 2.27.1, tximport, R 3.6,Bowtie2, IGV 2.4.0, Skewer version 0.2.2, BWA version 0.7.15, Deeptools version 2.5.7, awk version 4.0.2, Hisat2 version 2.1.0, StringTie version 1.2.3, Ballgown version 2.18.0, ChIPQC version 1.18.2, ggplot2 version 3.2.0, GenomicFeatures version 1.34.8, DESeq version 1.34.1, AnnotationDbi version 1.44.0, DiffBind version 2.10.0, Biobase version 2.42.0, GenomicRanges version 1.34.0, GenomeInfoDb version 1.18.2, BioVenn, SQuIRE, DESeq2.<br>The full pipeline including in-house scripts used for the analysis can be found in the following online repository (https://bitbucket.org/ADAC_UoN/adac0175-code/<br>Confocal Images were processed using Fiji Image J, Adobe Photoshop CS6 Version 13.0 x32, and Zeiss LSM 710 Zen 2012 Black Edition Service pack 5 imaging software.<br>Signal intensity data were plotted using GraphPad Prism 7.04.

For manuscripts utilizing custom algorithms or software that are central to the research but not yet described in published literature, software must be made available to editors and reviewers. We strongly encourage code deposition in a community repository (e.g. GitHub). See the Nature Research guidelines for submitting code & software for further information.

Data

Policy information about availability of data

All manuscripts must include a data availability statement. This statement should provide the following information, where applicable:

- Accession codes, unique identifiers, or web links for publicly available datasets
- A list of figures that have associated raw data
- A description of any restrictions on data availability

The rST and SZ deep sequencing data have been deposited in the EBI's European Nucleotide Achieve (ENA) (http://www.ebi.ac.uk/ena) under accession number PRJEB8358. MS source data for Figure 1b can be found in Supplementary Data 3. The confocal raw data and all other data supporting the conclusions of this study are available from the corresponding author upon reasonable request.

Field-specific reporting

Please select the one below that is the best fit for your research. If you are not sure, read the appropriate sections before making your selection.

☒ Life sciences ☐ Behavioural & social sciences ☐ Ecological, evolutionary & environmental sciences

For a reference copy of the document with all sections, see nature.com/documents/nr-reporting-summary-flat.pdf

Life sciences study design

All studies must disclose on these points even when the disclosure is negative.

Sample size	Samples size was chosen to ensure reproducibility of the results at affordable costs. Geneally, sample size was determined based on previosuly published studies and on experience and was equivalent to that is routinely used for any particular assay. Sample sizes are indicated separately for different experiments. At least 2 and typically 3 independent experiments were carried out for most of the assays. DIP was performed in two biologically independent experiments. We observed generally good correlation between the replicates for different experiments suggesting that choosen samples size was sufficient. No statistical methods were used to predetermine sample sizes.
Data exclusions	In case of experimental mistakes or occasional loss of samples/reagents, the experiment was discarded and repeated. Otherwise, no data were excluded from the analysis.
Replication	All experiments were replicated independently. All attempts at replication were successful.
Randomization	No randomization was required as the study was based on molecular and cellular biology techniques and did not involve allocation of experimental units across different treatment groups, no human subjects were involved.
Blinding	Blinding was not relevant to the study as the study was based on molecular and cellular biology techniques, the results were derived from objective quantitative methods. No subjective measurements were taken.

Reporting for specific materials, systems and methods

We require information from authors about some types of materials, experimental systems and methods used in many studies. Here, indicate whether each material, system or method listed is relevant to your study. If you are not sure if a list item applies to your research, read the appropriate section before selecting a response.

Materials & experimental systems

n/a	Involved in the study	
☐	☒	Antibodies
☒	☐	Eukaryotic cell lines
☒	☐	Palaeontology and archaeology
☐	☒	Animals and other organisms
☒	☐	Human research participants
☒	☐	Clinical data
☒	☐	Dual use research of concern

Methods

n/a	Involved in the study	
☐	☒	ChIP-seq
☒	☐	Flow cytometry
☒	☐	MRI-based neuroimaging

Antibodies

Antibodies used	Anti-5hmC mouse monoclonal (Active Motif, Catalog No: 39999, 1:5000 dilution), anti-5hmC rabbit polyclonal (Active Motif, Catalog No: 39791, 1:5000 dilution), anti-5mC mouse monoclonal (clone 33D3, Diagenode, catalogue number C15200081-100, 1:200 dilution), anti-5caC rabbit polyclonal (Active Motif, Catalog No: 61226, 1:500 dilution) and anti-5fC rabbit polyclonal

	(Active Motif, Catalog No: 61224, 1:500 dilution) primary antibodies were used for immunochemistry. Peroxidase-conjugated anti-rabbit secondary antibody (Dako) and the tyramide signal enhancement system (Perkin Elmer, 1:200 dilution, 2 min of incubation with tyramide) were employed for 5caC and 5hmC (rabbit polyclonal antibody) detection. 5hmC (mouse monoclonal antibody) and 5mC were visualized using 555-conjugated secondary antibody (Alexafluor, 1:400 dilution).
Validation	Anti-5-methylcytosine (5mC) antibody (clone 33D3, Diagenode, catalogue number C15200081-100) is widely used in immunostaining experiments (https://www.diagenode.com/en/p/5-mc-monoclonal-antibody-33d3-premium-100-ug-50-ul) and was validated for immunocytochemistry on human and mouse cells by the vendor and in other studies (e.g. Wheldon, L. M. et al. (2014) Cell Rep. 7, 1353-1361; Abakir, A., et al. (2016) J Vis Exp. 114).<br>Anti-5hmC mouse monoclonal (Active Motif, Catalog No: 39999, 1:5000 dilution), anti-5hmC rabbit polyclonal (Active Motif, Catalog No: 39791, 1:5000 dilution), anti-5caC rabbit polyclonal (Active Motif, Catalog No: 61226, 1:500 dilution) and anti-5fC rabbit polyclonal (Active Motif, Catalog No: 61224, 1:500 dilution) primary antibodies were validated for immunocytochemistry and DIP on human and mouse cells by the vendor and in other studies (e.g. Wheldon, L. M. et al. (2014) Cell Rep. 7, 1353-1361; Abakir, A., et al. (2016) J Vis Exp. 114).<br>https://www.activemotif.com/catalog/details/39999/5-hydroxymethylcytidine-antibody-mab-clone-59-1<br>https://www.activemotif.com/catalog/details/39791/5-hydroxymethylcytidine-antibody-pab<br>https://www.activemotif.com/catalog/details/61225/5-carboxylcytosine-antibody-5-cac-pab<br>https://www.activemotif.com/catalog/details/61223/5-formylcytosine-antibody-5-fc-pab

Animals and other organisms

Policy information about studies involving animals; ARRIVE guidelines recommended for reporting animal research

Laboratory animals	C57BL/6 wild type, CD1 wild type and CD1 GILZ Y/- male mice of various age were used for the study
Wild animals	The study did not involve wild animals.
Field-collected samples	The study did not involve samples collected from the field.
Ethics oversight	Experiments were performed in compliance with the UK and EU guidelines for the care 370 and use of laboratory animals. Animal procedures were subjected to local ethical review (Comite 371 d'Ethique pour l'Experimentation Animale, Universite Paris Descartes; registration number 372CEEA34.JC.114.12).

Note that full information on the approval of the study protocol must also be provided in the manuscript.

ChIP-seq

Data deposition

☒ Confirm that both raw and final processed data have been deposited in a public database such as GEO.

☒ Confirm that you have deposited or provided access to graph files (e.g. BED files) for the called peaks.

Data access links<br>*May remain private before publication.*	The rST and SZ deep sequencing data have been deposited in the EBI's European Nucleotide 516 Achieve (ENA) (http://www.ebi.ac.uk/ena) under accession number PRJEB8358.
Files in database submission	Cell type rST, Modification 5caC<br>Replicate 1: F_ca_1.bam<br>Replicate 2: F_ca_2.bam<br>Cell type rST, Modification 5mC<br>Replicate 1: F_m_1.bam<br>Replicate 2: F_m_2.bam<br>Cell type rST, Modification 5hmC<br>Replicate 1: F_h_1.bam<br>Replicate 2: F_h_2.bam<br>Cell type SZ, Modification 5caC<br>Replicate 1: S_ca_1.bam<br>Replicate 2: S_ca_2.bam<br>Cell type SZ, Modification 5mC<br>Replicate 1: S_m_1.bam<br>Replicate 2: S_m_2.bam<br>Cell type SZ, Modification 5hmC<br>Replicate 1: S_h_1.bam<br>Replicate 2: S_h_2.bam<br>peak files:<br>Peaks.rST_5caC.vs.RPKM.rST.tab.gz<br>Peaks.rST_5hmC.vs.RPKM.rST.tab.gz<br>Peaks.rST_5mC.vs.RPKM.rST.tab.gz<br>Peaks.SZ_5caC.vs.RPKM.rST.tab.gz<br>Peaks.SZ_5hmC.vs.RPKM.rST.tab.gz<br>Peaks.SZ_5mC.vs.RPKM.rST.tab.gz<br>Peaks.rST_5caC.vs.RPKM.SC.tab.gz<br>Peaks.rST_5hmC.vs.RPKM.SC.tab.gz<br>Peaks.rST_5mC.vs.RPKM.SC.tab.gz

Peaks.SZ_5caC.vs.RPKM.SC.tab.gz
Peaks.SZ_5hmC.vs.RPKM.SC.tab.gz
Peaks.SZ_5mC.vs.RPKM.SC.tab.gz

Genome browser session (e.g. UCSC)

The downstream visualization analysis was performed
using IGV, no web genome browser is available but an IGV session can be made available upon request.
The bed files have been deposited to the following online repository (https://bitbucket.org/ADAC_UoN/adac1075-bed-files/src).

Methodology

Replicates

2 Biological replicates were used for each DRIP experiment.

Sequencing depth

Sequencing depth information for SOLiD data is provided in Supplementary Fig. S5a.

Antibodies

anti-5hmC rabbit polyclonal (Active Motif, Catalog No: 39791, 1:5000 dilution), anti-5mC mouse monoclonal (clone 33D3, Diagenode, catalogue number C15200081-100, 1:200 dilution), anti-5caC rabbit polyclonal (Active Motif, Catalog No: 61226, 1:500 dilution) antibodies were used for DIP experiments.

Peak calling parameters

For SOLiD data, Highly modified (methylated) regions (HMRs, peaks) were first identified from alignment BAM files for each replicate sample using the peak-calling software MACS1.423. MACS1.4 parameters were: effective genome size = 1.87e+09, band width = 300, model fold = 10,30, pvalue cutoff = 1.00e-10. The input sample for the corresponding cell type was used as the background control. A p-value of 1e-10 was used to determine peaks. Highly confident peaks were subsequently identified for each sample by comparing the replicate peaks using the bioconductor package DiffBind. DiffBind parameters were: bScaleControl = TRUE, bParallel = TRUE, bCorPlot = TRUE, consensus = -DBA_REPLICATE. Differentially methylated (modified) regions (DMRs) between cell types were determined using the bioconductor package MEDIPS v1.14.025. MEDIPS parameters were: uniq = TRUE, extend = 300, shift = 0, ws = 500. Both replicate sample BAM files and the corresponding input sample BAM file for each cell type were used as input. A p-value of 0.01 was used to identify significant DMRs. Each peak or DMR was assigned to a gene if it was located within 1Kb of the gene coding sequence.

Data quality

Reads quality was assessed directly by the sequencing center using fastQC, the impact of each of the pull-downs on read distribution was assessed using Phantompeakqualtools and ChipQC was employed for assessing the peaks' genomic distribution and the quality of the data. Consensus peaks were defined using the dba.peakset() function to select for peaks overlapping in both replicates. Peaks were called with a q value of 0.01.

Software

Deep sequencing data were processed using open source software tools. The following packages were used: LifeScope (LifeTechnologies), MACS1.4, Samtools version 1.3.1, MEDIPS v1.14.0, LiftOver, Gviz, BEDtools, TopHat2, Htseq-count, Mfuzz, Partek Genomics Suite version 6.6 Gene 483 Set ANOVA, Picard Tools Dedup version 2.5.0, MACS2.1.1, PhantomPeakQualTools version - Feb 13, 2012, Bedtools version 2.27.1, tximport, R 3.6,Bowtie2, IGV 2.4.0, Skewer version 0.2.2, BWA version 0.7.15, Deeptools version 2.5.7, awk version 4.0.2, Hisat2 version 2.1.0, StringTie version 1.2.3, Ballgown version 2.18.0, ChIPQC version 1.18.2, ggplot2 version 3.2.0, GenomicFeatures version 1.34.8, DESeq version 1.34.1, AnnotationDbi version 1.44.0, DiffBind version 2.10.0, Biobase version 2.42.0, GenomicRanges version 1.34.0, GenomeInfoDb version 1.18.2, BioVenn.
The full pipeline including in-house scripts used for the analysis can be found in the following online repository (https://bitbucket.org/ADAC_UoN/adac0175-code/
